# Evaluating the effectiveness of psilocybin in alleviating distress among cancer patients: A systematic review

**DOI:** 10.1017/S147895152500032X

**Published:** 2025-04-22

**Authors:** Maria I. Lapid, Sandeep R. Pagali, Andrea L. Randall, Kristine A. Donovan, Carrie A. Bronars, Trevor A. Gauthier, Jonathan Bock, Samantha D. Lim, Elise C. Carey, Elizabeth Sokolowski, Angela M. Ulrich, Leslie C. Hassett, Simon Kung, Kevin J. Whitford, Kenneth R. Olivier, Stacy D. D’Andre

**Affiliations:** 1 Department of Psychiatry and Psychology, Mayo Clinic, Rochester, MN, USA; 2 Division of Community Internal Medicine, Geriatrics and Palliative Care, Mayo Clinic, Rochester, MN, USA; 3 Division of Hospital Internal Medicine, Mayo Clinic, Rochester, MN, USA; 4 Department of Pharmacy, Mayo Clinic, Rochester, MN, USA; 5 Department of Anesthesiology and Perioperative Medicine, Mayo Clinic, Rochester, MN, USA; 6 Alix School of Medicine, Mayo Clinic, Rochester, MN, USA; 7 Our Lady of Fatima College of Medicine, Valenzuela City, Metro Manila, Philippines; 8 Department of Quantitative Health Sciences, Mayo Clinic, Rochester, MN, USA; 9 Mayo Clinic Libraries, Mayo Clinic, Rochester, MN, USA; 10 Department of Radiation Oncology, Mayo Clinic, Rochester, MN, USA; 11 Department of Medical Oncology Mayo Clinic, Mayo Clinic, Rochester, MN, USA

**Keywords:** Anxiety, depression, existential distress, psychedelic, psychotherapy

## Abstract

**Objectives:**

Psychological and existential distress is prevalent among patients with life-threatening cancer, significantly impacting their quality of life. Psilocybin-assisted therapy has shown promise in alleviating these symptoms. This systematic review aims to synthesize the evidence on the efficacy and safety of psilocybin in reducing cancer-related distress.

**Methods:**

We searched MEDLINE, APA PsycINFO, Cochrane database, Embase, and Scopus from inception to February 8, 2024, for randomized controlled trials (RCTs), open-label trials, qualitative studies, and single case reports that evaluated psilocybin for cancer-related distress. Data were extracted on study characteristics, participant demographics, psilocybin and psychotherapy intervention, outcome measures, and results. Two authors independently screened, selected, and extracted data from the studies. Cochrane Risk of Bias for RCTs and Methodological Index for Non-Randomized Studies criteria were used to evaluate study quality. This study was registered with PROSPERO (CRD42024511692).

**Results:**

Fourteen studies met the inclusion criteria, comprising three RCTs, five open-label trials, five qualitative studies, and one single case report. Psilocybin therapy consistently showed significant reductions in depression, anxiety, and existential distress, with improvements sustained over several months. Adverse effects were generally mild and transient.

**Significance of results:**

This systematic review highlights the potential of psilocybin-assisted therapy as an effective treatment for reducing psychological and existential distress in cancer patients. Despite promising findings, further large-scale, well-designed RCTs are needed to confirm these results and address existing research gaps.

## Introduction

Cancer distress, characterized as a profound and multifaceted emotional turmoil, can severely impact patients’ ability to cope with their diagnosis and treatment, significantly diminishing their quality of life. The National Comprehensive Cancer Network defines cancer distress as “a multifactorial unpleasant experience of psychological (i.e., cognitive, behavioral, emotional), social, spiritual, and/or physical nature that may interfere with the ability to cope effectively with cancer, its physical symptoms, and its treatment. Distress extends along a continuum, ranging from common normal feelings of vulnerability, sadness, and fears to problems that can become disabling, such as depression, anxiety, panic, social isolation, and existential and spiritual crisis (Smith et al. 2018; Vehling and Kissane 2018).” Studies have shown that approximately 40–50% of cancer patients experience moderate to clinically significant levels of distress at some point during their illness trajectory (Carlson et al. 2012; Mehnert et al. 2018). This distress often stems from the emotional burden of a cancer diagnosis itself, treatment side effects, fear of recurrence, previous mental health history financial concerns, lack of social support, and existential concerns (Herschbach et al. 2020; Ikhile et al. 2024; Lewandowska et al. 2020). In advanced cancer stages, existential distress becomes particularly pronounced, manifesting as feelings of hopelessness, meaninglessness, and intense fear of death (Rodin et al. 2009). Additionally, cancer distress can profoundly impact both patients and their families’ quality of life, emotional health, roles within the family, and ability to engage in treatment (Caruso et al. 2021; Ferrell and Wittenberg 2017; Hodges et al. 2005; Teo et al. 2023). Addressing this distress is crucial for improving the overall well-being and quality of life of patients with cancer, as well as their psychological resilience and ability to cope with the disease (Teo et al. 2019).

Interventions for cancer distress encompass a range of therapeutic approaches (e.g., psychoeducation, cognitive behavior therapy, relaxation training) that target the emotional and physical changes faced by individuals diagnosed with cancer. Research regarding the effectiveness of these interventions demonstrates low to moderate effect sizes (Faller et al. 2013; Park et al. 2019; Sanijda et al. 2018). This highlights the need to explore novel therapeutic interventions that may better address the multifaceted nature of cancer distress.

Psilocybin is a naturally occurring tryptamine found in several mushroom species. It is rapidly hydrolyzed in the liver to the psychoactive compound psilocin. It has been investigated for its potential therapeutic benefits in treating a variety of psychological conditions. Psilocybin acts on serotonin receptors in the brain, particularly the 5-HT2A receptor, which is believed to play a role in mood regulation and cognitive flexibility (Nichols 2016). “Classical psychedelics,” including psilocybin, lysergic acid diethylamide, N,N-dimethyltryptamine, and mescaline, share this mechanism of action but also have complex pharmacologic action at several other receptor sites as well (Kwan et al. 2022; Nichols 2016; Reiff et al. 2020). Proposed mechanisms for the effects of psilocybin include neurobiological mechanisms, with several potential signaling cascades implicated, which may promote neuroplasticity, as well as psychological mechanisms, including increased cognitive flexibility and the influence of mystical experiences (Doss et al. 2021; Griffiths et al. 2016; Ko et al. 2022; Ross et al. 2016).

Reported effects of psilocybin include perceptual distortions, labile emotions, euphoria, ego dissolution (a decrease in self-referential thinking), and hallucinations (Kwan et al. 2022; Nichols 2016). Classical psychedelics can also cause mystical experiences, which encompass such features as ineffability, transcendence of time and space, universal interconnectedness, and a deeply felt positive mood, among others (Ko et al. 2022; Nichols 2016). Such experiences are thought to partially mediate the therapeutic benefits of psychedelics (Reiff et al. 2020). In non-cancer patients with depression, psilocybin has shown promise in reducing symptoms of depression and anxiety, contributing to improved emotional and psychological well-being (Carhart-Harris et al. 2016, 2018). For patients with cancer who often face the dual challenges of managing a life-threatening illness and coping with the associated psychological burden, psilocybin has been shown to decrease symptoms of depression, anxiety, and existential distress, and increase overall well-being and life satisfaction (Griffiths et al. 2016; Ross et al. 2016). This therapeutic potential makes psilocybin a promising candidate for addressing the unmet needs of cancer patients, offering a holistic approach that addresses both the emotional and existential dimensions of their distress.

Given the substantial burden of psychological and existential distress in cancer patients and the promising preliminary evidence suggesting the potential efficacy of psilocybin-assisted therapy, there is a clear rationale for conducting a systematic review on this topic. This review will consolidate existing findings, identify consistent outcomes, and highlight areas needing further investigation. This systematic review aims to fill this gap by providing a thorough evaluation of the current evidence, including both quantitative and qualitative data, to better understand the therapeutic potential and safety profile of psilocybin in this context. Additionally, the insights gained from this systematic review will inform and guide the development of our own clinical trial aimed at evaluating psilocybin therapy for distress in cancer patients, with the ultimate goal of improving interventions for managing distress in this population.

## Methods

### Search strategy and selection criteria

The protocol for this systematic review was registered with the International Prospective Register of Systematic Reviews, PROSPERO (CRD42024511692). The reporting of this systematic review was guided by the standards of the Preferred Reporting Items for Systematic Review and Meta-Analysis (PRISMA) Statement (Moher et al. 2009).

A comprehensive search of several databases was performed on February 9, 2024 and updated on August 26, 2024. Results were limited to English Language. No date limits for the search were applied. Databases searched (and their content coverage dates) were Ovid MEDLINE(R) (1946+ including epub ahead of print, in-process, and other nonindexed citations), Ovid Embase (1974+), Ovid Cochrane Central Register of Controlled Trials (1991+), Ovid Cochrane Database of Systematic Reviews (2005+), Ovid APA PsycInfo (1967+) and Scopus via Elsevier (1970+).

The search strategies were designed and conducted by a medical librarian with input from the study investigators. Controlled vocabulary supplemented with keywords was used. The strategies listing all search terms used and how they were combined are available in the supplemental material.

### Data extraction and quality assessment

Randomized controlled trials (RCTs), open-label or case series studies, and single case reports that met the inclusion criteria were selected. Extracted study information included study characteristics (authors, year of publication, location), participant demographics (age, gender, diagnosis), details of psilocybin (formulation, dose, frequency, duration, other treatment parameters) and psychotherapy interventions, study methodology (study design, randomization, control condition, blinding), outcome measures (primary and secondary outcomes, tools or instruments used), and results (distress and other psychological measures/scores before and after intervention, effect sizes, statistical significance). Covidence software was utilized for review, data extraction, and reporting. Two authors independently screened titles and abstracts, selected articles for full-text review, and extracted data. Two reviewers independently assessed the quality of RCTs using the Cochrane Risk of Bias Tool (Schunemann et al. 2019) and evaluated non-randomized studies using the Methodological Index for Non-Randomized Studies (MINORS) criteria (Slim et al. 2003). Any disagreements were resolved through discussion until a consensus was reached.

## Results

### Search results

**Figure 1.**
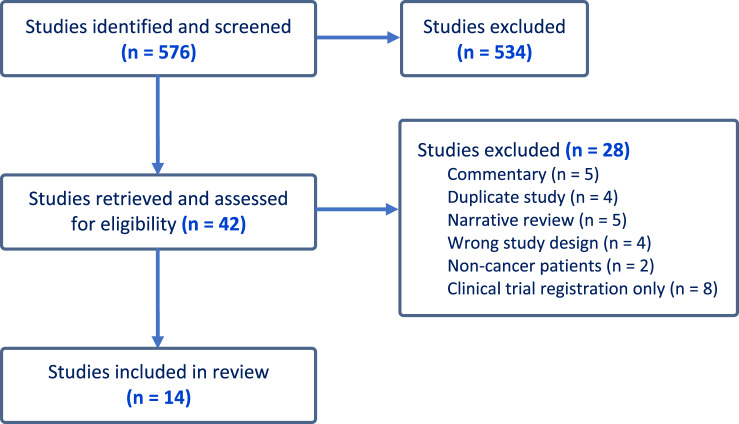
PRISMA flow diagram.

A total of 576 abstracts and titles were initially identified from database searches. After removing duplicates and irrelevant studies, 42 studies were assessed for eligibility, out of which 28 studies were excluded. Fourteen studies met the inclusion criteria and were included in the systematic review, as shown in the PRISMA flow diagram (Fig. 1).

### Characteristics of included studies

#### RCTs

Three RCTs collectively demonstrated the efficacy and safety of psilocybin in reducing anxiety and depression in patients with life-threatening cancer (Table 1). Griffiths et al. (2016) conducted a double-blind cross-over trial with 51 patients, revealing that a high dose of psilocybin led to substantial and sustained decreases in depression and anxiety, with effects lasting up to six months. Grob et al. (2011) conducted a pilot within-subject study with 12 patients, reporting significant reductions in anxiety and mood improvements following psilocybin treatment that lasted for several months. Ross et al. (2016) found that a single dose of psilocybin significantly reduced anxiety and depression symptoms in 29 patients, with improvements persisting for six months and notable enhancements in quality of life and emotional well-being. Adverse effects across these studies were generally mild and transient, including blood pressure elevations, headaches, nausea, and temporary anxiety, with no serious adverse events reported.

**Table 1.** Summary of included studies

Study, author (year), country	Study design	Study population	Sample size, % female, mean age years ± SD (or SEM), (range)	Psilocybin regimen	Placebo/control	Outcome measures	Findings	Adverse events
Griffiths et al. (2016)	RCT	Cancer with anxiety/mood symptoms, DSM-IV GAD, MDD, dysthymic disorder	Total: 51 49% 56.3 (1.4)	Single high dose 22 mg/70 kg for 49 subjects, 30 mg/70 kg for 1 subject	Single low dose 3 mg/70 kg for 12 subjects, 1 mg/70 kg for 38 subjects	Depression: GRID- HAM-D-17; Anxiety: HAM-A; secondary measures (BDI, HADS, STAI, POMS, BSI, MQOL, LOT-R, LAP-R Death Acceptance, Death Transcendence, Purpose in Life Test, LAP-R Coherence)	Substantial and enduring decreases in depression, anxiety, death anxiety; improved quality of life; effects sustained at 6 months.	BP elevation, nausea, anxiety, headache.
USA	Double-blind cross-over		High dose: 26 50% 56.5 (1.8)					No SAE.
			Low dose: 25 48% 56.1 (2.3)					
Grob et al. (2011)	RCT	Cancer with DSM-IV anxiety disorders	12 50%	Single moderate dose (0.2 mg/kg)	Single dose niacin (250 mg)	Safety: BP, HR, temp; Depression: BDI; Mood: POMS; Anxiety: STAI; Psychiatric symptoms: BPRS; Perception: 5D-ASC	Reductions in depression (6 months) and anxiety (3 months); no impact on pain.	HR and BP elevations, diastolic BP reduction after niacin.
USA	Within-subject double-blind placebo-controlled		56.0 ± 10.7 (36–58)					Drug order apparent to subjects and investigators.
Ross et al. (2016)	RCT	Cancer with DSM IV-TR ASD, GAD, anxiety due to cancer, adjustment disorder	29 62 %	Single moderate dose (0.3 mg/kg)	Single dose niacin (250 mg)	Safety: BP, HR;	Rapid and sustained anxiolytic and antidepressant effects sustained for 7–8 months; existential distress reduced; improved spiritual wellbeing and quality of life; decreased demoralization and hopelessness.	No SAE.
USA	Blinded crossover		56.28 ± 12.93 (22–75)			Depression: BDI, HADS; Anxiety: STAI		
Agin-Liebes et al. (2020)	Cohort study	Cancer with DSM IV-TR ASD, GAD, anxiety due to cancer, adjustment disorder	15 60%	Single moderate dose (0.3 mg/kg)	Single dose niacin (250 mg)	Depression: HADS-A, HADS-D, HADS-T, BDI-II; Anxiety: STAI-S, STAI-T; Demoralization; Hopelessness; Death Anxiety; Meaning/peace; Faith	Large significant reductions in anxiety, depression, hopelessness, demoralization, death anxiety; improvements in spiritual well-being up to 4.5 years after psilocybin.	None.
USA	Long-term follow-up from Ross et al. 2016 RCT		53 ± 15.5 (25–73)					
Agrawal et al. (2023)	Open label	Cancer with DSM-V MDD	30 70%	Single dose 25 mg	N/A	Depression: MADRS, Quick, Maudsley; Anxiety: STAI-S and STAI-T, HAM-A; Pain: VAS; Demoralization: DS-II; Disability: SDS, EQ-5D-5 L	Group psilocybin-assisted therapy was safe and feasible. Substantial symptom reduction in depression and anxiety and enhanced group cohesion.	Nausea, headache.
USA			56 ± 12 (30–78)					No SAE.
Anderson et al. (2020)	Open label	Gay-identified cis-gender men with demoralization, cancer survivors	18 0% (all males)	Single dose 0.3 mg/kg for cohort 1 and 0.36 mg/kg po for cohorts 2, 3	N/A	Safety: BP and HR; Suicidality: C-SSRS, ChEQ; Anxiety and Depression: SAHD, MoCA, STAI-S and STAI-Tk AUDIT, DUDIT; Demoralization: DS-II; CESD-R, CGI-S, MQoL-R	Demonstrated feasibility, safety, efficacy (reducing demoralization) of psychedelic-assisted group therapy in serious medical illness. Improved mood and emotional well-being.	Hypertension, nausea, anxiety, paranoia, hallucinations, dissociation. Unexpected reactions of PTSD flashback, methamphetamine relapse.
USA			59.2 ± 4.4 (50–66)					
Lewis et al. (2023)	Open label	Cancer with DSM-5 depressive disorder	12	Single dose 25 mg	N/A	Depression: HAMD-17; FACIT-Sp, MEQ-30	Clinically substantial reductions in depression up to 26 weeks.	Nausea, headache, hypertension – all required medications.
USA			66.7% 48.2 ± 11.5					No SAE.
Shnayder et al. (2023)	Open label	Cancer with DSM IV MDD	30	Single dose 25 mg	N/A	Psycho-Social-Spiritual wellbeing: NIH-HEALS Connection, Reflection/Introspection, and Trust/Acceptance	Psilocybin-assisted therapy facilitated psycho-social-spiritual growth.	Nausea, headache, tearfulness, anxiety, euphoria, fatigue, impairment of psychomotor functioning.
USA			70% 56 ± 12 (30–78)					
Patchett-Marble et al. (2022)	Single case report	Stage IV small cell lung cancer with existential and psychological distress	1	Single high dose *Psilocybe cubensis* (5 g) tea	N/A	GAD-7, PHQ-9, MQOL-R, MEQ-30, Persisting Effects Questionnaire	Immediate, substantial, sustained improvement in psychological and existential distress and quality of life, sustained at 4 months.	Not reported.
USA			100%					
			54					

5D-ASC – 5-Dimension Altered States of Consciousness; ASD – Acute Stress Disorder; AUDIT – Alcohol use Disorder Identification Test; BDI – Beck Depression Inventory; BP – Blood pressure; BPRS – Brief Psychiatric Rating Scale; BSI – Brief Symptom Inventory; CESD-R – Center for Epidemiological Studies Depression Scale-Revised; CGI-S – Clinical Global Impressions Scale, severity of illness; C-SSRS – Columbia Suicidality Severity Rating Scale; DS-II – Demoralization Scale II; DSM – Diagnostic and Statistical Manual of Mental Disorders; DUDIT – Drug Use Disorders Identification Test; EQ-5D-5 L – EuroQOL-5-dimension-5-level scale; FACIT-Sp – Functional Assessment of Chronic Illness Therapy-Spiritual Well-Being Scale, version 4; GAD – Generalized Anxiety Disorder; GAD-7 – Generalized Anxiety Disorder-7 item; GRID-HAMD-17 – GRID-Hamilton Rating Scale for Depression; HADS – Hospital Anxiety and Depression Scale; HADS A – Hospital Anxiety and Depression Scale Anxiety; HADS D – Hospital Anxiety and Depression Scale Depression; HADS T – Hospital Anxiety and Depression Scale Total; HAM-A with SIGH-A – Hamilton Anxiety Rating Scale assessed with Structured Interview Guide for the Hamilton Anxiety Scale; HR – Heart rate; LAP-R – Life Attitude Profile – Revised; MADRS – Montgomery-Asberg Depression Scale; Maudsley – Maudsley Visual Analog Scale; MDD – Major depressive disorder; MEQ-30 – Death Transcendence Scale – Mystical Experience Questionnaire; MoCA – Montreal Cognitive Assessment; MQOL – McGill Quality of Life; Pain VAS – Visual Analog Scale; PHQ-9 – Patient Health Questionnaire-9 item; POMS – Profile of Mood States; PTSD – Posttraumatic stress disorder; QIDS-SR – Quick Inventory of Depressive Symptomatology-Self Report; QUICK – Quick Inventory of Depressive Symptomatology-Self Report; RCT – Randomized controlled trial; SAE – Serious adverse events; SAHD – Schedule of Attitudes towards Hastened Death; SD – Standard deviation; SDS – Sheehan Disability Scale; STAI – State-Trait Anxiety Inventory; STAI-S – State-Trait Anxiety Inventory State Anxiety; STAI-T – State-Trait Anxiety Inventory Trait Anxiety; SEM – Standard Error of the Mean; Temp – Temperature.

#### Open-label studies

Five open-label trials investigated psilocybin-assisted therapy for cancer-related distress, showing promising outcomes in psychological, social, and spiritual well-being (Table 1). In a long-term follow-up study from an RCT conducted by Ross et al. (2016), Agin-Liebes and their team found sustained improvements in psychiatric and existential distress up to 4.5 years post-psilocybin treatment (Agin-Liebes et al. 2020). Agrawal et al. (2023) conducted psilocybin-assisted group therapy in cancer patients with major depressive disorder, noting significant reductions in depression and anxiety and enhanced group cohesion. Anderson et al. (2020) studied psilocybin-assisted group therapy in demoralized older men who are long-term AIDS survivors and cancer survivors, reporting improved mood and emotional well-being. Reported adverse events during dosing included hypertension, nausea, anxiety, paranoia, hallucinations, and transient thought disorder. Lewis et al. (2023) conducted the Hopkins-Oxford Psychedelics Ethics (HOPE; A Pilot Study of Psilocybin Enhanced Group Psychotherapy in Patients with Cancer) pilot study, focusing on psilocybin-enhanced group psychotherapy for cancer patients, which resulted in significant distress alleviation and enhanced group support, with adverse effects such as hypertension, nausea, and headache requiring medications. Shnayder et al. (2023) found that psilocybin-assisted therapy significantly improved psycho-social-spiritual well-being in cancer patients, highlighting its potential as a holistic therapeutic approach.

#### Single case report

Patchett-Marble et al. (2022) presented a case report detailing the use of psilocybin mushrooms to treat psychological and existential distress in a patient with lung cancer. The patient experienced significant relief from anxiety, depression, and existential distress after a single session, with improvements in quality of life and emotional well-being sustained at 4 months.

#### Qualitative studies

Five qualitative studies provide insights into patients’ experiences undergoing psilocybin-assisted therapy for cancer-related distress, complementing the quantitative results from RCTs and open-label trials. (Table 2) Beaussant et al. (2023) reported high acceptability and significant emotional and psychological benefits from psilocybin-assisted group therapy in patients with cancer and major depressive disorder. Belser et al. (2017), Swift et al. (2017), and Malone et al. (2018) derived their data from the previously cited Ross RCT. Belser et al. (2017) reported emotional and spiritual experiences that led to lasting reductions in existential distress and enhanced well-being. Swift et al. (2017) highlighted transformative emotional and existential experiences, providing insights into long-term psychological benefits. Malone et al. (2018) described the individual experiences of four cancer patients, reporting profound personal insights, emotional breakthroughs, and a renewed sense of meaning. Lewis et al. (2023) provided observations from the HOPE trial, focusing on group-format psychedelic-assisted therapy. Patients emphasized the therapeutic value of shared experiences and group support, contributing to significant emotional relief and enhanced social connectedness.

**Table 2.** Summary of qualitative studies

Study Author (year) Country	Study population	Sample size % Female Mean age years ± SD (range)	Methodology	Findings	Comments
Beaussant et al. (2023)	Cancer with DSM IV MDD	28	Semi-structured interviews guided by the conceptual framework of acceptability.	High acceptability of group and simultaneous sessions. Group experienced enhanced safety and efficacy, sense of connection and belonging, deepened meaning of experience through transcendence and compassion.	Data from Agrawal et al. (2023) open label trial.
USA		67.8% 56.2 ± 11.4 (30–78)			
Belser et al. (2017)	Cancer with DSM IV-TR ASD, GAD, anxiety due to cancer, adjustment disorder	13	Semi-structured interviews employing interpretative phenomenological analysis (IPA).	Themes included relational embeddedness, a range of emotions, music enhancing the experience, meaningful visuals, wisdom gained, revised life priorities, and desire to repeat the experience with psilocybin.	Data from Ross et al. (2016) RCT
USA		46% 50 ± 15.77 (22–69)			
Lewis et al. (2023) USA	Cancer with DSM − 5 depressive disorder	10	Qualitative survey questionnaires on the group-format design, with free-form written comments. The 5 questions were rated from 1 to 5 (1-strongly disagree, 2-disagree, 3-neutral, 4-agree, 5-strongly agree): “I felt like the group format psilocybin session worked well and maximized my individual therapeutic response; I felt an increased connection to other group members in virtue of having a group format psilocybin session and having contact with their individual processes; I felt like the group psilocybin session was a natural extension of the group process initiated through the preparatory sessions; I felt like having a communal music track worked well; I would have preferred to have an individual music track with headphones.”	Positive impressions of the group-based psilocybin protocol. Considerations to combine Supportive Expressive Group Therapy principles with psilocybin education, emphasize safety and confidentiality, maintain openness in emotional exploration, and acknowledge varied participant experiences. Music was played through speakers rather than individual headphones to emphasize the group nature, however, results were mixed as some desired individual over communal music experience.	Data from Lewis et al. (2023) open label (HOPE) trial.
Malone et al. (2018)	Cancer with DSM IV-TR ASD, GAD, anxiety due to cancer, adjustment disorder	4	Description of clinical course using quantitative measures, qualitative interviews, narratives, and clinical notes.	Each experience was unique and personal. However, personal narratives had similar themes of self-compassion and love, death acceptance, and past trauma memories. The subjective experiences helped participants understand and meet their own spiritual and psychological needs.	Data from Ross et al. 2016 RCT.
USA		50% 20–60 s			
Swift et al. (2017)	Cancer with DSM IV-TR ASD, GAD, anxiety due to cancer, adjustment disorder	13	Semi-structured interviews employing interpretative phenomenological analysis (IPA).	Ten themes identified related to cancer, death, and healing. Participants discussed anxiety and trauma from cancer and a perceived lack of emotional support. They described the immersive and distressing effects of the psilocybin session, leading to reconciliations with death, acknowledgment of cancer’s role in life, and emotional detachment from cancer. Spiritual or religious interpretations were common, with psilocybin therapy facilitating a reconnection to life, reclaiming of presence, and increased confidence in facing cancer recurrence.	Data from Ross et al. 2016 RCT.
USA		85.7% 50 ± 15.77 46%			Combined with Belser et al. (2017), although Swift et al. (2017) focuses on cancer, death, and healing from emotional distress.

ASD – Acute stress disorder; DSM – Diagnostic and Statistical Manual of Mental Disorders; GAD – Generalized Anxiety Disorder; MDD – Major depressive disorder; HOPE – A Pilot Study of Psilocybin Enhanced Group Psychotherapy in Patients with Cancer.

### Psychotherapy interventions

The psychotherapy interventions across the reviewed studies varied in conceptual basis and structure, incorporating individual and group therapy formats (Table 3). Grob et al. (2011) utilized supportive therapy principles, focusing on individual preparation and emotional support during dosing, with limited details on the integration phase. The Griffiths et al. (2016) and Ross et al. (2016) studies combined supportive-expressive and existential psychotherapy approaches, emphasizing individual preparation and integration through several hours of meetings to discuss meaningful life aspects and process the psilocybin experience. In contrast, Agrawal et al. (2023) adapted the COMPASS Pathways model for a group format, integrating psychoeducation and supportive techniques with both group and individual sessions for preparation and integration. Anderson et al. (2020) employed a model based on Brief Supportive Expressive Group Therapy (SEGT), emphasizing emotional expression and support, with a mix of individual and group sessions to prepare for and integrate the psilocybin experience. Lewis et al. (2023) and Shnayder et al. (2023) incorporated SEGT principles combined with psychoeducation, focusing on trust-building, coping strategies, and processing experiences through group and individual sessions.

**Table 3.** Summary of psychotherapy interventions

Study author (year) country	Conceptual basis of psychotherapy	Preparatory sessions	Dosing sessions	Integration sessions	Total duration
**RCT**					
Grob et al. (2011)	Rooted in supportive therapy principles, emphasizing preparation and emotional support during dosing.	Meetings to review purpose and intention, treatment goals, structure of sessions, and potential emotional reactions.	Conducted in a hospital clinical research unit with pleasing decoration and comfortable environment.	Discussion of post-session subjective experiences from cognitive, affective, and psychospiritual perspective.	6-h dosing session.
USA		Establish rapport, review significant life issues, and address current concerns.	Participants lie in bed, with eye shades and pre-selected music. Checked on by staff each hour.	Completion of rating instruments.	Limited details on integration phase
Griffiths et al. (2016)	Integrates elements of supportive-expressive therapy, existential psychotherapy, and psychoeducation.	Establish rapport, prepare participant for psilocybin session, discussion of meaningful aspects of participant’s life.	Conducted in living-room-like environment.	The integration phase included follow-up meetings to help participants process and integrate their experiences. The meetings focused on exploring new thoughts and feelings, making sense of the experiences, and applying insights to daily life. This comprehensive approach ensured that participants were adequately prepared, supported throughout the experience, and able to derive lasting benefits from the therapy.	Mean duration of sessions were 7.9 h for preparation with an average of 3 sessions; 7 h for dosing sessions; and an average of 2.5 h of integration post-dosing.
USA			Participants lie on couch, with eye mask and headphones with music. Attention focused on inner experiences, with no further directions. Monitors were “nondirective and supportive, and encouraged participants to trust, let go and be open to the experience.”		
Ross et al. (2016)	Drawn from palliative care, existential, psychoanalytic therapy, and transpersonal psychology.	Discussion of meaningful life aspects and preparation for psilocybin experience.	Conducted in a living-room-like environment.	Post-dosing sessions focused on discussion of thoughts and feelings that were novel that arose during dosing sessions.	Average of 3 preparatory sessions totaling 7.9 h.
USA			Participants lie on couch, with eye shades and pre-selected music.		Post dosing therapy was day 1 after each dose, then final session 6 weeks after 2nd dosing.
			Therapists present for 8-h session for psychological and medical support.		Total study participation was 9 months (mean 253 days).
			During sessions, monitors were nondirective and supportive, and they encouraged participants to “trust, let go and be open” to the experience.		
			At the end of dosing session, participants discuss subjective experience to consolidate the memory of the experience and begin post-integration.		
**Open-label**					
Agin-Liebes et al. (2020)	From Ross 2016 RCT	NA	NA	NA	NA
USA					
Agrawal et al. (2023)	COMPASS Pathways model adapted for group format, integrating psychoeducation and supportive techniques.	Two preparatory sessions:	Group participants used eyeshades and listened to music.	Two integration sessions with group and individual components.	8 h of preparation and integration therapy over 8 weeks.
USA		Information about psilocybin, coping strategies, therapeutic alliance.	Therapist stayed in adjacent room.	Focus on processing experiences during dosing and exploring their meaning and impact.	4.25 h of individual support.
		A 2-h group and individual therapy. Group therapy involved psychoeducation and building trust among participants. Individual therapy focused on discussing concerns, setting intentions for session, learning techniques to manage anxiety, and experiential engagement.	The therapeutic approach was non-directive, with group goals of ensuring psychological safety, maintaining each one’s attention on the present moment, and processing emotional state.	Therapists refrain from making interpretations or suggesting solutions; they provide psychoeducation to support the integration and apply the experience to daily lives.	3.75 h of group support.
Anderson et al. (2020)	Modeled after Brief Supportive Expressive Group Therapy (SEGT), emphasizing emotional expression and support.	Individual psychotherapy to build rapport and trust and prepare for psilocybin and group therapy.	Focus on “here and now” processing, group support, and expressing emotions.	Post-dosing discussion of psilocybin experience and generate meaning from the experience that can be applied to day-to-day lives.	90 min per session.
USA				Group therapy focused on palliative care-based existential psychotherapy that prioritized “here and now processing, mutual support, detoxifying death and emotional expression.”	
				Each session started and ended with 5-min breathing exercises and meditations on self-compassion and mindfulness.	
Lewis et al. (2023)	Based on supportive-expressive group therapy principles, combined with psychoeducation on psilocybin.	3 group preparatory sessions (120 min each, 90 min group time, 30 min individual).	Dosing in a communal space with semiprivate bays.	3 group integration sessions (120 min each, 90 min group time, 30 min individual).	3 group preparatory sessions (120 min each), 1 dosing session, and 3 group integration sessions (120 min each).
USA		Supportive-expressive group process with psychoeducation about psilocybin effects and dosing logistics.	Non-directive, supportive approach with communal music playlist.	Focus on sharing experiences, integrating insights into personal narratives, and exploring ongoing integration techniques (journaling, engagement, mindfulness).	Total duration was 3 weeks for each cohort of 4 participants.
Shnayder et al. (2023)	Based on supportive therapy principles, focusing on coping strategies and therapeutic alliance.	Two preparatory sessions:	Group participants used eyeshades and listened to music.	Group and individual therapy, aimed to integrate psychological material accessed during dosing and exploring their meaning and impact.	Total of 8 visits during the 8-week study period.
USA		A 2-h visit to introduce psilocybin treatment, coping strategies, breathing exercises, and establish therapeutic alliance. A 2-h group and individual therapy. Group therapy involved psychoeducation and building trust among participants. Individual therapy focused on discussing concerns, setting intentions for session, learning techniques to manage anxiety, and experiential engagement.	The therapeutic approach was non-directive, with group goals of ensuring psychological safety, maintaining each one’s attention on the present moment, and processing emotional state.	Therapists refrain from making interpretations or suggesting solutions; they provide psychoeducation to support the integration and apply the experience to daily lives.	

NA = Not applicable.

### Risk of bias assessment

The qualitative assessment of the included RCT studies, evaluated using the Cochrane Risk of Bias Tool, and the open-label studies, assessed with the MINORS criteria, are presented graphically and in tabular form in the supplemental material. Overall, the quality of the studies was found to be modest. In RCTs, blinding of the psilocybin arm would be very difficult, as expected.

## Discussion

The findings from this systematic review highlight the potential of psilocybin as a therapeutic intervention for depression, anxiety, and overall mood improvement in patients with cancer. This therapy may help reduce depression and anxiety while improving overall quality of life and emotional well-being. These benefits have been observed across RCTs and open-label trials, indicating a therapeutic potential that warrants cautious further exploration.

### Ethical considerations

The consensus from the HOPE Working Group emphasized the need to consider all the ethical dimensions of psilocybin-assisted therapy (Jacobs et al. 2024). The HOPE workshop discussed the contributions and rights of Indigenous communities with long histories of using psychedelic substances and the need to engage with these communities respectfully and equitably for ethical research and clinical practices. The workshop also highlighted the need for a precautionary approach to advancing scientific understanding. Despite promising safety profiles, the long-term and contextual risks of psilocybin are not fully understood, necessitating comprehensive and representative research efforts. This aligns with our systematic review findings that while psilocybin therapy shows potential benefits, more diverse and extensive studies are needed to generalize results across different populations and settings.

### Addressing safety concerns and ensuring appropriate use

While the potential benefits of psilocybin-assisted therapy are encouraging, it is crucial to acknowledge concerns related to the use of psychedelics, particularly in the context of rising recreational use. Psychedelics, including psilocybin, are not without risks, and their misuse and abuse can lead to adverse outcomes. However, the studies included in our systematic review involved psilocybin administered in structured clinical settings with comprehensive support from trained therapists. This controlled environment is essential for ensuring safety and maximizing therapeutic benefits. Appropriate use and rigorous monitoring are key to mitigating risks. This includes thorough patient screening, careful dosing, close supervision during and after the psychedelic experience, and extensive training for therapists. These measures help prevent potential misuse and ensure that therapy is safe and effective (Schlag et al. 2022). Patients with a history of, or strong family history of, psychosis or bipolar disorder should be excluded from these treatments (Johnson et al. 2008).

Despite the promise of psychedelic-assisted therapies, there is a critical need for improved risk-benefit assessments and broader, more inclusive studies to safely integrate these therapies into modern healthcare (Bradberry et al. 2022). Addressing potential psychiatric risks, such as transient effects and worsening mood or psychosis in susceptible individuals, is essential. These challenging experiences, however, may lead to improved outcomes (Griffiths et al. 2016). Integrating psychedelic therapy education into psychiatric and clinical psychology training programs may also help address potential access bottlenecks as well as ensure providers are trained in comprehensive risk-benefit evaluations for patients. Providing strong patient support and establishing appropriate regulatory frameworks will be essential for safely and effectively implementing these therapies (Bradberry et al. 2022).

### Importance of psychotherapy in psilocybin treatment

A critical aspect of the efficacy of psilocybin in these studies is its use in conjunction with psychotherapy. The therapeutic setting, including preparation, dosing, and integration sessions, is vital in maximizing benefits and mitigating risks. Psilocybin-assisted psychotherapy provides a structured environment where patients can safely and meaningfully process their experiences. The supportive presence of trained therapists helps guide patients through challenging emotions, facilitating deeper insights and emotional breakthroughs. The combination of psilocybin with psychotherapy is crucial for achieving therapeutic outcomes, ensuring the psychedelic experience is integrated into the patient’s broader psychological context.

The studies in our systematic review demonstrate the flexibility of psilocybin-assisted psychotherapy, utilizing individual and group therapy formats to provide comprehensive support throughout the therapeutic process. The choice between individual and group therapy formats reflects different therapeutic goals and logistical considerations, with group therapy offering opportunities for shared experiences and mutual support. In contrast, individual therapy provides tailored, one-on-one attention to address personal concerns. Incorporating spiritual, existential, and psychological components, psilocybin-assisted psychotherapy shows promise in significantly reducing psychological distress in patients with cancer. Still, it requires careful monitoring and culturally sensitive care to maximize benefits and mitigate risks (Palitsky et al. 2023).

### Meaningfulness of small improvements in distress

In the context of life-threatening illnesses such as cancer, even small improvements in psychological and existential distress can be profoundly meaningful. Patients with terminal diagnoses often experience high levels of anxiety, depression, and existential dread, which can severely impact their quality of life (Krikorian et al. 2012). The ability to alleviate these symptoms through psilocybin-assisted therapy, even modestly, can significantly enhance a patient’s emotional and psychological well-being. For many patients with cancer, a reduction in distress can translate into an improved ability to enjoy meaningful interactions with loved ones, better engagement in daily activities, and a greater sense of peace as they navigate their illness.

### Limitations and areas for future research

While the current body of evidence is promising, it has limitations. The relatively small sample sizes, modest study quality, and the open-label design of several studies introduce potential biases. In RCTs, patients can often tell whether they have received psilocybin or a placebo due to the noticeable effects of the psychedelic, which can affect the study blinding and outcomes. Future research should focus on larger, multi-center RCTs to confirm these findings and further explore the combined therapeutic effects of psilocybin and the associated therapy. Additionally, investigating the long-term impact of psilocybin-assisted therapy on different cancer types and stages will provide a more comprehensive understanding of its applicability. Expanding research to include diverse populations and settings will help generalize the findings. There is also potential in exploring synthesized molecules that do not have the psychedelic effect, as suggested by Dr. Charles Raison, a prominent researcher in the field of psychedelic studies.

### Clinical implications

Psilocybin-assisted therapy has shown the potential to offer a rapid and sustained reduction in symptoms of depression and anxiety among cancer patients, which is particularly relevant in palliative care settings. The reported long-term benefits suggest that a limited number of psilocybin sessions could lead to enduring improvements in distress. However, these findings are preliminary and should be interpreted with caution until validated by larger, more rigorous studies.

## Conclusion

This systematic review is unique in that it comprehensively examines the effects of psilocybin on cancer-related distress, highlighting its potential as a therapeutic option. Psilocybin-assisted therapy may help alleviate psychological and existential distress in cancer patients, with benefits observed across multiple studies. While these findings are encouraging, they should be approached with cautious optimism. More large-scale, well-designed RCTs are needed to confirm these results and address existing research gaps. Integrating psilocybin-assisted therapy into clinical practice under appropriate supervision and with rigorous monitoring could offer a viable treatment option for managing distress in cancer patients. Additionally, the findings from this systematic review will inform and guide the development of our own clinical trial to evaluate psilocybin therapy for distress in cancer patients, aiming to improve interventions for managing distress in this population.

## Supporting information

Lapid et al. supplementary material Lapid et al. supplementary material
