# Supplementary material for: Evaluating the effectiveness of psilocybin in alleviating distress among cancer patients: A systematic review
Source: Palliat Support Care. 2025 Apr 22;23:e99. doi: 10.1017/S147895152500032X (PMC13166453; doi:10.1017/S147895152500032X)
Supplement: Lapid et al. supplementary material [file S147895152500032Xsup001.docx]

**Evaluating the Effectiveness of Psilocybin in Alleviating Distress Among Cancer Patients: A Systematic Review and Meta-analysis**

**Supplemental Material**

**Figure S1**. Cochrane risk of bias for randomized controlled studies


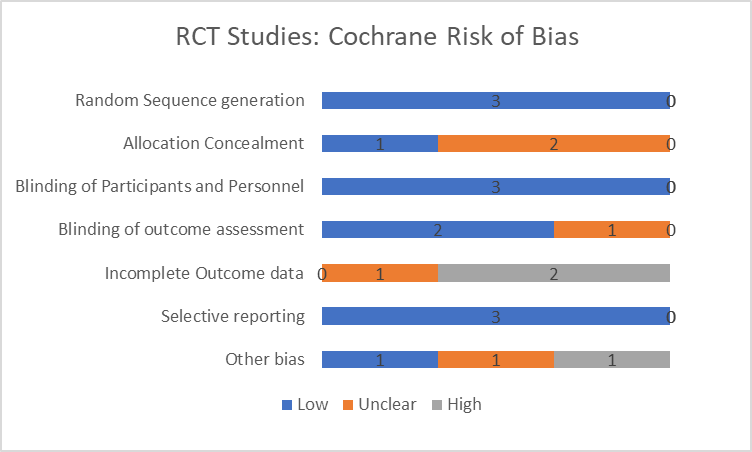


**Figure S2**. Methodological Index for Non-Randomized Studies (MINORS) risk of bias for open label studies


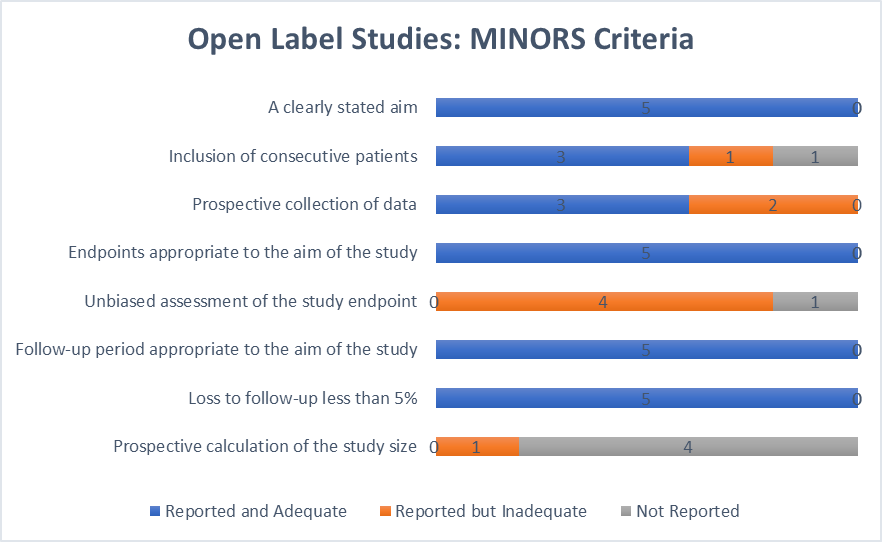


**Table S1**. Cochrane risk of bias details for randomized controlled studies

| **RCT Studies: Cochrane Risk of Bias** | **Random Sequence Generation** | **Allocation Concealment** | **Blinding of Participants and Personnel** | **Blinding of Outcome Assessment** | **Incomplete Outcome Data** | **Selective Reporting** | **Other Bias** |
| --- | --- | --- | --- | --- | --- | --- | --- |
| Griffiths 2016 | Low | Unclear | Low | Unclear | Unclear | Low | High |
| Grob 2011 | Low | Unclear | Low | Low | High | Low | Unclear |
| Ross 2016 | Low | Low | Low | Low | Unclear | Low | Low |

**Table S2**. Methodological Index for Non-Randomized Studies (MINORS) risk of bias details for open label studies

| **Open label Studies (MINORS criteria)** | **A clearly stated aim** | **Inclusion of consecutive patients** | **Prospective collection of data** | **Endpoints appropriate to the aim of the study** | **Unbiased assessment of the study endpoint** | **Follow-up period appropriate to the aim of the study** | **Loss to follow-up less than 5%** | **Prospective calculation of the study size** |
| --- | --- | --- | --- | --- | --- | --- | --- | --- |
| Agin-Liebes 2020 | 2 | 2 | 2 | 2 | 1 | 2 | 2 | 1 |
| Agrawal 2023 | 2 | 2 | 2 | 2 | 1 | 2 | 2 | 0 |
| Anderson 2020 | 2 | 0 | 1 | 2 | 0 | 2 | 2 | 0 |
| Lewis 2023 | 2 | 1 | 1 | 2 | 1 | 2 | 2 | 0 |
| Shnayder 2023 | 2 | 2 | 2 | 2 | 1 | 2 | 2 | 0 |

**Search Strategies**

**OVID**

Database(s): Ovid MEDLINE(R) 1946 to Present and Epub Ahead of Print, In-Process & Other Non-Indexed Citations and Ovid MEDLINE(R) Daily, APA PsycInfo 1967 to February Week 2 2024, EBM Reviews - Cochrane Central Register of Controlled Trials January 2024, EBM Reviews - Cochrane Database of Systematic Reviews 2005 to February 7, 2024, Embase 1974 to 2024 February 08. Search updated on August 26, 2024.
Search Strategy:

| **#** | **Searches** |
| --- | --- |
| 1 | exp distress/ |
| 2 | psychological stress/ or psychological distress/ or Stress, Psychological/ |
| 3 | exp anxiety/ |
| 4 | exp fear/ |
| 5 | depression/ |
| 6 | suffering/ |
| 7 | *Palliative Care/ |
| 8 | *Terminal Care/ |
| 9 | *Hospice Care/ |
| 10 | *Critical Illness/ |
| 11 | (distress* or stress or angst or demorali* or suicidal or suicidality or fear* or anxiety or depression or depressed or anguish* or suffer* or "well-being" or "quality-of-life" or "symptom-burden").ti,ab,kf,tw. |
| 12 | (hospice or palliative or "critically ill" or "end-of-life" or dying or "life-threatening").ti,ab. |
| 13 | or/1-12 |
| 14 | exp Neoplasms/ |
| 15 | (cancer* or neoplas* or paraneoplas* or tumor* or tumour* or leukemia* or carcinoma* or lymphoma* or astrocytoma* or glioma* or adenoma* or carcinoid* or sarcoma* or ostesarcoma* or histiocytoma* or craniopharyngioma* or ependymoma* or chordoma* or "Chronic Myeloproliferative Disorder*" or craniopharyngioma* or "Mycosis Fungoide*" or "Sézary Syndrome*" or Esthesioneuroblastoma* or melanoma* or retinoblastoma* or histeocytoma* or "gestational trophoblastic disease*" or histiocytos* or burkitt* or Macroglobulinemia* or Mesothelioma* or neuroblastoma* or Papillomatos* or paraganglioma* or "pheochromocytoma* multiple myeloma*" or blastoma* or Rhabdomyosarcoma* or nonmelanoma* or metasta*).ti,ab,hw,kf. |
| 16 | or/14-15 |
| 17 | (Psilocybin or psilocybine or bufotenine or elymoclavine or hallucinogen* or "lysergic acid" or lysergide or mebufotenin or mescaline or midomafetamine or mushroom* or psilocine* or psilocybine or salvinorin or tenocyclidine).ti,ab,hw,tw,kf. |
| 18 | Psilocybin/ or psilocybine/ or psychedelic agent/ or bufotenine/ or elymoclavine/ or lysergic acid/ or lysergide/ or mebufotenin/ or mescaline/ or midomafetamine/ or tenocyclidine/ |
| 19 | 17 or 18 |
| 20 | 13 and 16 and 19 |
| 21 | limit 20 to english language [Limit not valid in CDSR; records were retained] |
| 22 | (conference abstract or conference review or editorial or erratum or note or addresses or autobiography or bibliography or biography or blogs or comment or dictionary or directory or interactive tutorial or lectures or legal cases or legislation or news or newspaper article or patient education handout or periodical index or portraits or published erratum or video-audio media or webcasts).mp. or conference abstract.st. |
| 23 | 21 not 22 |
| 24 | (exp animals/ or exp nonhuman/) not exp humans/ |
| 25 | ((alpaca or alpacas or algae* or amphibian or amphibians or animal or animals or antelope or armadillo or armadillos or avian or baboon or baboons or bats or beagle or beagles or bee or bees or bird or birds or bison or bovine or buffalo or buffaloes or buffalos or "c elegans" or "Caenorhabditis elegans" or camel or camels or canine or canines or canis or carp or cats or catfish or cattle or chamaeleo* or chameleon* or chick or chicken or chickens or chicks or chimp or chimpanze or chimpanzees or chimps or cow or cows or "D melanogaster" or "dairy calf" or "dairy calves" or deer or dog or dogs or donkey or donkeys or drosophila or "Drosophila melanogaster" or duck or duckling or ducklings or ducks or equid or equids or equine or equines or feline or felines or ferret or ferrets or finch or finches or fish or flatworm or flatworms or fox or foxes or frog or frogs or "fruit flies" or "fruit fly" or "G mellonella" or "Galleria mellonella" or geese or gerbil or gerbils or goat or goats or goose or gorilla or gorillas or groundhog or groundhogs or hamster or hamsters or hare or hares or heifer or heifers or horse or horses or iguana or iguanas or insect or insects or jellyfish or kangaroo or kangaroos or kitten or kittens or "laboratory animal*" or lagomorph or lagomorphs or lamb or lambs or lemur or lemurs or lemuridae or llama or llamas or macaque or macaques or macaw or macaws or marmoset or marmosets or mice or minipig or minipigs or mink or minks or monkey or monkeys or mouse or mule or mules or muskrat or muskrats or nematode or nematodes or newt or newts or octopus or octopuses or orangutan or "orang-utan" or orangutans or "orang-utans" or oxen or parrot or parrots or pig or pigeon or pigeons or piglet or piglets or pigs or porcine or primate or primates or poultry or quail or rabbit or rabbits or rat or rats or reptile or reptiles or rodent or rodents or ruminant or ruminants or salmon or sheep or shrimp or slug or slugs or swine or tamarin or tamarins or tilapia or tilapias or toad or toads or trout or urchin or urchins or vole or voles or waxworm or waxworms or weasel or weasels or wolf or wolves or worm or worms or wrass* or xenopus or "zebra fish" or zebrafish) not (human or humans or patient or patients)).ti,ab,hw,kw. |
| 26 | (rat or rats or mice or mouse or murine or pig or pigs or porcine or swine or dog or dogs).ti. |
| 27 | or/24-26 |
| 28 | 23 not 27 |
| 29 | remove duplicates from 28 |

**SCOPUS**

| 1 | TITLE-ABS-KEY ( psilocybin OR psilocybine OR bufotenine OR elymoclavine OR hallucinogen* OR "lysergic acid" OR lysergide OR mebufotenin OR mescaline OR midomafetamine OR mushroom* OR psilocine* OR psilocybine OR salvinorin OR tenocyclidine ) |
| --- | --- |
| 2 | TITLE-ABS-KEY (distress* or stress or angst or demorali* or suicidal or suicidality or fear* or anxiety or depression or depressed or anguish* or suffer* or "well-being" or "quality-of-life" or "symptom-burden") |
| 3 | TITLE-ABS-KEY ( ( hospice OR palliative OR "critically ill" OR "end-of-life" OR dying OR "life-threatening" cancer* OR neoplas* OR paraneoplas* OR tumor* OR tumour* OR leukemia* OR carcinoma* OR lymphoma* OR astrocytoma* OR glioma* OR adenoma* OR carcinoid* OR sarcoma* OR ostesarcoma* OR histiocytoma* OR craniopharyngioma* OR ependymoma* OR chordoma* OR "Chronic Myeloproliferative Disorder*" OR craniopharyngioma* OR "Mycosis Fungoide*" OR "Sézary Syndrome*" OR esthesioneuroblastoma* OR melanoma* OR retinoblastoma* OR histeocytoma* OR "gestational trophoblastic disease*" OR histiocytos* OR burkitt* OR macroglobulinemia* OR mesothelioma* OR neuroblastoma* OR papillomatos* OR paraganglioma* OR "pheochromocytoma* multiple myeloma*" OR blastoma* OR rhabdomyosarcoma* OR nonmelanoma* OR metasta* ) ) |
| 4 | 1 and 2 and 3 |
| 5 | INDEX(embase) OR INDEX(medline) OR PMID(0* OR 1* OR 2* OR 3* OR 4* OR 5* OR 6* OR 7* OR 8* OR 9*) |
| 6 | 4 not 5 |
| 7 | LIMIT-TO ( LANGUAGE , "English" ) |
